# Digital Games for Type 1 and Type 2 Diabetes: Underpinning Theory With Three Illustrative Examples

**DOI:** 10.2196/games.3930

**Published:** 2015-03-18

**Authors:** Maged N Kamel Boulos, Shauna Gammon, Mavis C Dixon, Sandra M MacRury, Michael J Fergusson, Francisco Miranda Rodrigues, Telmo Mourinho Baptista, Stephen P Yang

**Affiliations:** ^1^The Alexander Graham Bell Centre for Digital HealthMoray College UHIUniversity of the Highlands and IslandsElginUnited Kingdom; ^2^Ayogo Health Inc.Vancouver, BCCanada; ^3^Department of Diabetes and Cardiovascular ScienceDivision of Health Research, Centre for Health ScienceUniversity of the Highlands and IslandsInvernessUnited Kingdom; ^4^Faculdade de PsicologiaUniversidade de LisboaLisbonPortugal; ^5^Health Promotion & WellnessSUNY OswegoOswego, NYUnited States

**Keywords:** diabetes mellitus, video games, self-care

## Abstract

Digital games are an important class of eHealth interventions in diabetes, made possible by the Internet and a good range of affordable mobile devices (eg, mobile phones and tablets) available to consumers these days. Gamifying disease management can help children, adolescents, and adults with diabetes to better cope with their lifelong condition. Gamification and social in-game components are used to motivate players/patients and positively change their behavior and lifestyle. In this paper, we start by presenting the main challenges facing people with diabetes—children/adolescents and adults—from a clinical perspective, followed by three short illustrative examples of mobile and desktop game apps and platforms designed by Ayogo Health, Inc. (Vancouver, BC, Canada) for type 1 diabetes (one example) and type 2 diabetes (two examples). The games target different age groups with different needs—children with type 1 diabetes versus adults with type 2 diabetes. The paper is not meant to be an exhaustive review of all digital game offerings available for people with type 1 and type 2 diabetes, but rather to serve as a taster of a few of the game genres on offer today for both types of diabetes, with a brief discussion of (1) some of the underpinning psychological mechanisms of gamified digital interventions and platforms as self-management adherence tools, and more, in diabetes, and (2) some of the hypothesized potential benefits that might be gained from their routine use by people with diabetes. More research evidence from full-scale evaluation studies is needed and expected in the near future that will quantify, qualify, and establish the evidence base concerning this gamification potential, such as what works in each age group/patient type, what does not, and under which settings and criteria.

## Introduction

### Challenges of Managing Blood Glucose Control in Children and Adolescents With Type 1 Diabetes

Type 1 diabetes is a chronic condition generally manifested in childhood or the early teenage years. While genetic and environmental factors are involved, the precipitating factors are unknown. Autoimmune destruction of the insulin-producing cells in the pancreas means that daily administration of insulin is necessary. A high degree of self-management and regular monitoring is required to achieve and sustain the level of glycemic control necessary, and to offset visual problems, nerve damage, renal impairment, and other long-term complications [1].

Children with type 1 diabetes have to monitor their blood glucose and diet, administer insulin, and participate in physical activity during school hours, which represent a large portion of a child’s waking hours [2]. This situation can feel overwhelming and burdensome, if not stigmatizing, as the child tries to mingle with his or her peers who do not have diabetes.

Adolescence is a time of transition from childhood to adulthood, when physical and psychosocial changes can have an impact on the management of long-term conditions developed in earlier childhood. Deterioration in diabetes control is often experienced in adolescence [3] due to myriad factors, including increased insulin requirements related to growth and other hormonal changes [4]. This deterioration is also due to variable compliance with treatment regimens as the young person gradually becomes more independent of parental involvement and takes ownership of glucose monitoring and insulin administration. This may be compounded by social environments and peer pressure where experimentation with risk-taking behaviors occurs, for instance, around alcohol, illicit drugs, and sexual activity—evidence suggests that adolescents with diabetes may be more vulnerable to conform to peer norms [5]. Furthermore, suboptimal glycemic control may also be a deliberate choice, in particular the desire to avoid hypoglycemia in social settings. Psychosocial difficulties that can surface in adolescence, including depression and low self-esteem, may in addition contribute to poor self-management and indifferent control. Problems with weight gain, especially in girls, can trigger disordered eating and have significant adverse effects on diabetes control, given that insulin omission is an adaption used commonly to enhance weight loss [6].

Thus, there is a greater challenge facing the young person with type 1 diabetes during the adolescent years, highlighting the need to motivate and develop enhanced coping skills to confront the added burden of diabetes compared with their nondiabetic peers. Detection and recognition of these factors is endorsed in the health care professional approach to management of adolescents with type 1 diabetes. Guidelines have been developed on best practice, including educational provision, decision support, and transition to adult services [7,8]. However, education provided by conventional approaches appears to have less effect in promoting self-care in adolescents [9]. As well, while techniques such as motivational interviewing to aid self-management through behavior change may be helpful for adults, this is less likely to be successful with adolescents who have diabetes [10]. Generating educational opportunities that optimize self-care behavior through problem solving and target setting have been advocated for this group through the use of modern technology [11]. Communication technologies may increase the frequency of contact between the patient and health care professional, but it remains unclear whether this results in improved outcomes [12].

There is, therefore, a pressing need to find innovative solutions at scale that encourage children and young people with diabetes to continually engage with glucose monitoring and therapy compliance during the transition phase to emerging adulthood. Solutions should assist decision support, be personalized, be responsive to individual needs, and demonstrate acceptability alongside measurable outcomes in increased self-management, quality of life, and crucially, maintenance of good glycemic control.

### Challenges in Access to Information and Education to Aid Self-Management of Type 2 Diabetes

Type 2 diabetes constitutes about 90% of diabetes globally. It is a progressive condition related to the body’s ineffective use of insulin. Genetics, age, and ethnicity are important risk factors, however, excess body weight and physical inactivity contribute significantly to the development of type 1 diabetes. It is often managed by lifestyle interventions initially, but with a gradual stepwise increase in oral therapies leading to injectable therapy for a large proportion of sufferers in order to achieve an acceptable level of glycemic control. The ultimate goal must be prevention of type 2 diabetes through improved prediction of those at risk and participation in lifestyle and behavior changes that will reduce or delay the onset of type 2 diabetes. However, optimizing quality of life through prevention of diabetes-related long-term complications, such as blindness, renal failure, amputations, and the high level of cardiovascular morbidity related to poor glycemic control, is essential to reduce direct and indirect health care costs associated with diabetes comorbidities [13,14].

Despite the high prevalence of type 2 diabetes, access to information, education, and support can be fragmented across communities. Contact with trained health care professionals can provide for some of these needs, although implementing and sustaining advice from health care professionals can be difficult [15]. We should aspire to reducing people’s dependence on health professionals and increasing their sense of control and well-being [16].

In addition, many people with diabetes feel isolated and want to connect with others who can empathize with their experiences. This connection is needed at the time of diagnosis when feeling overwhelmed by the new “label” and the information to be assimilated, or later when struggling with the burden of living with a chronic disease, when new therapies are introduced, or when problems arise. Participation in information exchange through social or peer-to-peer interaction may be an important strategy to aid self-management and thus promote compliance with lifestyle measures and medical interventions that will culminate in the maintenance of good glycemic control.

Devices and apps that combine technology with entertainment and social interaction could prove a significant advance in the acquisition of skills to sustain self-reliance for individuals with type 2 diabetes at varying stages of their condition and to benefit biomedical, psychosocial, and lifestyle measures.

### Digital Games for Diabetes

The Internet and mobile health offer tools to enhance self-care through access to education and information and general support for people living with diabetes [17]. There are further benefits to be realized for health care providers through reduction in hospital attendance and admission, travelling times for staff and patients, and time away from family or employment [18]. It is important that digital interventions meet expectations and realize optimal outcomes during use. Human factors research and patient codesign of apps and content are essential to achieve these goals [19].

Digital games are an important class of digital interventions in diabetes, made possible by the Internet and a good range of affordable mobile devices (ie, mobile phones and tablets) available to consumers today. Gamifying disease management can help children, adolescents, and adults with diabetes to better cope with their lifelong disease [20,21]. Gamification and social in-game components can be used to motivate players/patients and positively change their behavior and lifestyle, for example, help them develop the good habit of regular self-measurement of blood glucose [11,22]. Games would offer rewarding experiences in the form of “achievements” that can be shared with other players, progress points, and/or in-game virtual currency rewards—that can be spent to “buy” in-game power-ups—to help achieve all of this.

Moreover, video exergames—games involving physical exercise and burning calories, for example, those played using Kinect [23] or the global positioning system (GPS) functionality of mobile phones [24]—can help obese patients with type 2 diabetes become more active and fit. Games can also be used to educate and train health care professionals about various aspects of diabetes [25]. However, these uses are not the focus of this paper.

In this paper, we provide three short illustrative examples of mobile and desktop game apps and platforms for type 1 diabetes—one example of a game for mobile devices (Monster Manor)—and type 2 diabetes—two examples of games for mobile and desktop platforms (Empower) and for mobile devices and Facebook (HealthSeeker). We provide these examples to support our discussion of the potential of gamification and digital game mechanics as adherence tools in the management of diabetes, and how these mechanics might work, including the underpinning psychological mechanisms for behavior change.

The featured game apps were developed by Ayogo Health, Inc, based in Vancouver, BC, Canada. They target different age groups with different needs (ie, children with type 1 diabetes vs adults with type 2 diabetes). They are each briefly described, and we also provide some highlights of the received users’ feedback. We do not provide a detailed user evaluation of these game examples, as this was not the focus of this paper, although we do hint in our text at some previous and ongoing evaluation studies involving these games—the results of some of these evaluation studies were still under embargo at the time of writing in September 2014. In all the mentioned evaluation exercises, Ayogo and its partners followed the established ethical procedures and norms when dealing with patients, and parents of children, with diabetes.

It should be noted that this paper is not meant to serve as an exhaustive review of all digital game genres available for type 1 and type 2 diabetes. There are many other digital game offerings for diabetes besides the three illustrative examples below. For example, the Juvenile Diabetes Research Foundation (UK) offers a couple of online, Web browser-based, type 1 diabetes games aimed at children and teenagers [26]. In addition, Boehringer Ingelheim and Eli Lilly & Company have an educational digital game exclusively for type 2 diabetes called Complications Combat—online [27] and for iOS [28].

### The Ayogo Model

The Ayogo Model used in designing the game apps presented in this paper is an evidence-based approach to designing games and gamified apps to build self-efficacy—the belief in one’s own ability to manage and demonstrate self-control—among people managing chronic illness and diabetes in particular. This approach blends Design Thinking, evidence from cognitive and behavioral psychology, and video game design.

Design Thinking is a method of rapid, empathic problem solving. It begins with primary user research and observation to uncover insights about the root needs of the player (or patient). For example, players/patients with type 1 diabetes can be expected to provide different insights than players/patients with type 2 diabetes. A Design Thinking process would be expected to unveil different root needs that, in turn, shape different player behavior goals for persons managing type 1 or type 2 diabetes.

The Ayogo Model also incorporates into its solutions an understanding of cognitive biases and related user interaction with Web and mobile technology. For example, the model asserts that designs should seek to reduce choice complexity, leverage defaults for decision making, tap into social/group/herd decision making, and harness loss aversion.

Furthermore, the model looks to video game design for excellent examples of persuasive technology, and applies these techniques to the health challenge of managing diabetes. The resulting designs are most akin to casual mobile video games. The three main elements that social video games, and games in general, share with the Ayogo Model approach to developing health apps are:

1. Narrative. Providing context and reassurance to players with chronic illness, narrative reassures the player that the information they will find in the game/app is relevant to them. Playful and delightful narrative and visual elements can reframe illness as a challenge that can be overcome. Narrative and iconography that provide common cultural references help players/patients recognize that the app is *for them.* The player may write their own goals into the visual narrative of the game.

2. Progression. The game or app breaks difficult health goals into incremental, progressively difficult small steps. Through common game elements such as levels, headers, experience points (XP), collections, and visualization, the player receives feedback and rewards for progressing toward their goal through the app.

3. Social interaction. Based on analysis of games such as HealthSeeker and Empower (both of which are described below), the Ayogo Model found evidence that designs that maximize opportunities to generate “incoming messages” to players will sustain player engagement longer than games that do not. The success of this approach is also seen in the phenomenal success of Facebook and other social media sites. This can be done in games by leveraging the social graph, designing for reciprocal obligations, gifting, and by sending scripted messages of encouragement and empathy toward others. Social features are used to address players’ desires to connect with others facing a shared adversity.

The theoretical underpinnings of the Ayogo Model are further discussed later in this paper, following the presentation of the illustrative examples.

### Illustrative Examples of Games That Used the Ayogo Model

#### Monster Manor (Type 1 Diabetes)

**Figure 1:**
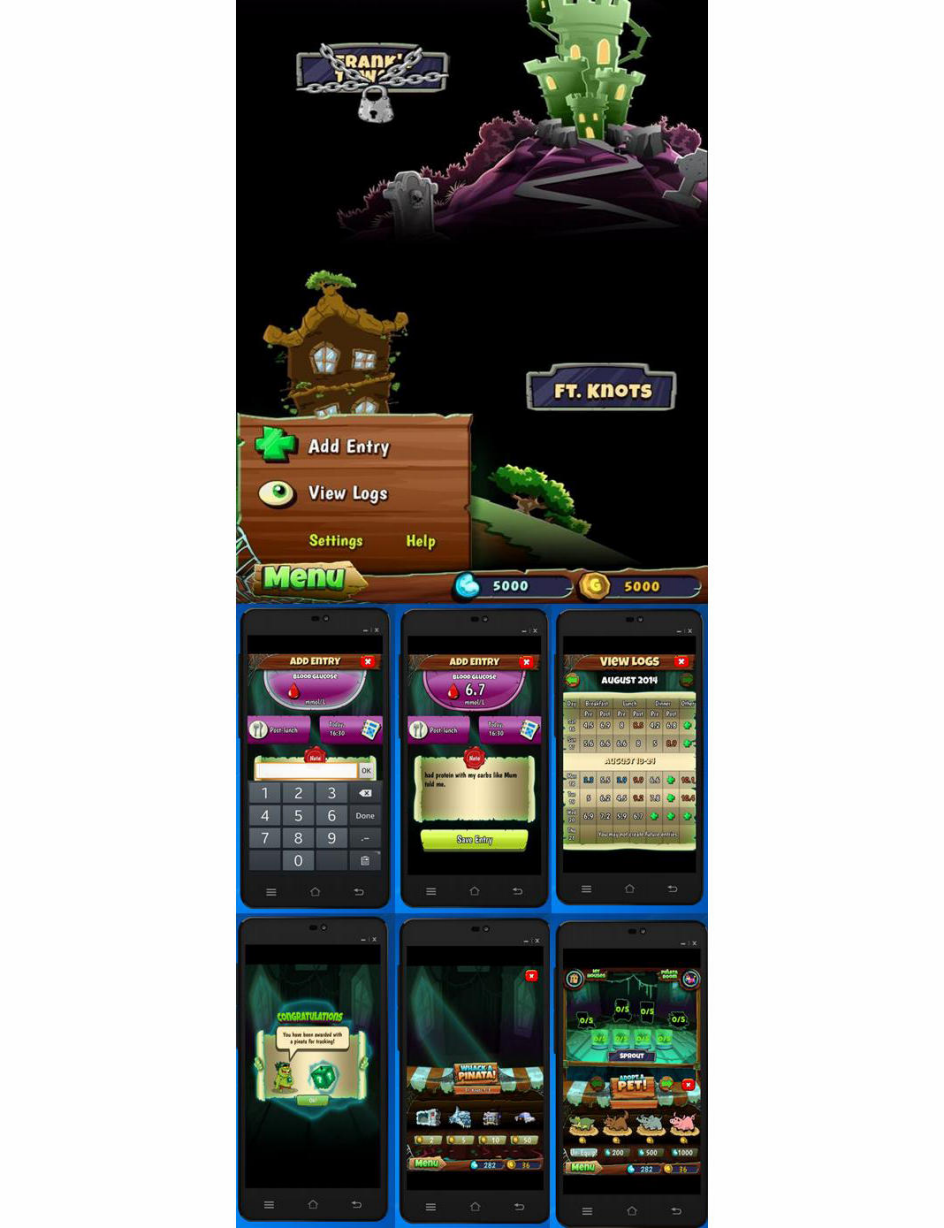
Screenshots of Monster Manor. Children can input their blood glucose measurements using an on-screen numeric pad and earn virtual coins to buy various in-game items that are essential for progression through the game.

Ayogo created Monster Manor (Figure 1) in collaboration with Sanofi to help families who struggle with the challenge of managing type 1 diabetes [29,30]. Children between ages 6 and 10 with type 1 diabetes are expected to take on increasing responsibility for testing and logging their own blood glucose. By incorporating a casual-play collecting game, Monster Manor provides a fun and rewarding experience for those children who struggle with this growing responsibility. Testing and logging blood glucose within the game’s built-in tracker generates positive feedback to keep children engaged in this crucial aspect of their self-care. Users were involved in the design of the game. Interviews with parents whose children tested early and successive versions of the app, and who were asked about their and their child’s feelings regarding Monster Manor, were internally gathered and analyzed by Ayogo for the purposes of informing and refining the game’s user experience (UX) and design.

Although it is theoretically easy to cheat in Monster Manor about blood glucose measurements (or even input unrealistic values) to get in-game rewards, it should be stressed that the game has a pretty narrow focus and audience, and is primarily intended to encourage *supervised* children to engage with their logging tools. For a variety of reasons, regulatory and otherwise, Monster Manor is not interpreting the data that children enter in any way. The app developers could certainly have gone another step and built (complex and expensive) integrations with hardware blood glucose meters (cf, Bayer DIDGET working on the Nintendo DS and hard-tying the rewards to actual measurements via a blood glucose meter [31]). However, this would have probably quadrupled the budget for the project and the marginal utility of doing that would not have quadrupled given the app’s goals and narrow audience (ie, kids supervised by their parents). Typically, the software is installed on the parent’s device (eg, iPad) and used there, so ultimately the parent can monitor everything the child does and the values they enter. Having the child work independently on their own handheld device would change the scope quite a bit.

#### Empower (Type 2 Diabetes)

Empower is a gamified digital health platform that helps patients to habituate a doctor-prescribed program of medication introduction and lifestyle change over 30 to 90 days. It motivates and supports patients as they adopt a new curriculum of doctor-prescribed behaviors specific to their condition. It does this by delivering educational content that connects to real-world healthy activities. Empower is designed to employ peer-support and reciprocity through social gameplay to motivate action and sustain repeat engagement.

A diabetes-focused iteration of Empower was play-tested over several weeks with results gathered by a third-party market research group (n=27). All players were women aged 35 to 60, who had been diagnosed with diabetes within the last 12 months (most diagnosed within the last 6 months). None of the players had a preexisting social relationship. The full results of the test are covered by a nondisclosure agreement made at the time of writing (September 2014), but the test confirmed that the app was building self-efficacy as players valued peer support from others with diabetes, and social features sustained repeat engagement with educational content.

Adults with type 2 diabetes want to connect with others who can empathize with their experiences. Therefore, in Empower, players/patients can share tips, participate in on-topic discussions, and provide peer-to-peer encouragement. Field testing of Empower with guided, but free, text commenting (1100 comments) resulted in no negative comments posted, no adverse drug events reported, and no posts flagged by users for content moderation.

Empower achieves effectiveness through progressive mastery. Newly diagnosed patients may feel overwhelmed by all the information that comes with diagnosis. Empower provides the most important information to patients in actionable, repeatable, progressive, small steps that build confidence in one’s ability to manage one’s condition. In-game quizzes give patients feedback on their mastery of the health curriculum.

Game mechanics, such as levels, collections, experience points, user interface (UI), progression, summary charts, among others, help patients visualize how far they have come.

Empower uses narrative—cumulative episodes in a story—to connect the content and game elements with the target audience. The narrative helps patients to recognize themselves in the game and allows them to find meaning in using the app.

Players enjoyed unlocking narratives that were suspenseful (eg, cliff hangers in a series of related stories), educational (eg, choose your own adventure with players controlling the decision-making path to a positive or negative health outcome), and entertaining (eg, relatable, humorous, or emotionally engaging).

It takes time to change behavior, so Empower is designed to lightly engage players over a period of months. The stickiness of social engagement and the addictiveness of variable rewards are key ways in which Empower may achieve long-term engagement.

**Figure 2:**
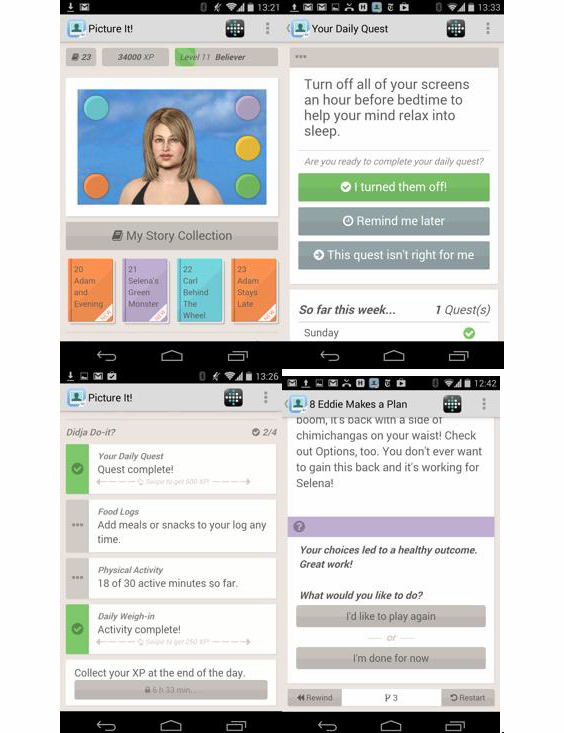
Screenshots of Picture It! with fitbit integration.

Empower is currently being used as a foundation for the following two apps:

1. Type 2 Travelers is a desktop and mobile app developed in collaboration with Merck Sharp and Dohme that is intended to support adults with type 2 diabetes. It is based on the Empower platform and features players’ avatars and in-game virtual currency [32].

2. Picture It! (Figure 2) is a mobile version of Empower with biometric integration (fitbit [33]), but without peer-to-peer support, for patients preparing for bariatric surgery through weight loss and new habit formation. It is currently being evaluated in an A/B test by an integrated health care network in California (n=60). Pilot-test results were under embargo at the time of writing (September 2014).

#### HealthSeeker (Type 2 Diabetes)

**Figure 3:**
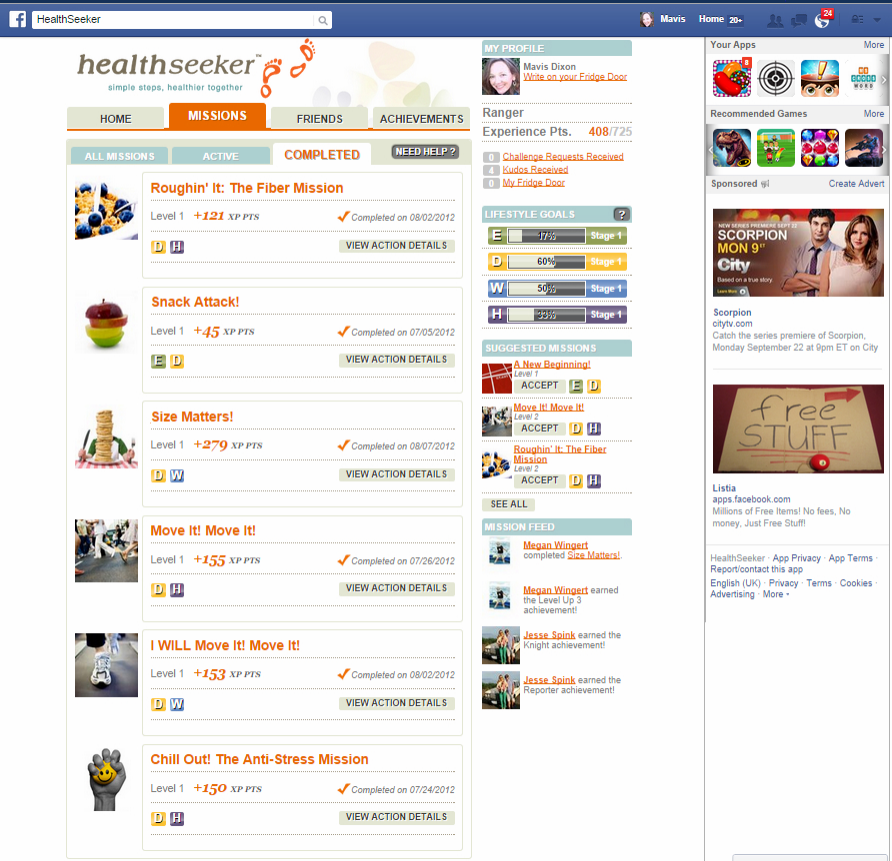
Screenshot of HealthSeeker on Facebook.

HealthSeeker [34], released in 2010, was the first-of-its-kind health game on Facebook to help people with diabetes improve their health through lifestyle changes (Figure 3). The game is also available as an app for Android and iOS devices. HealthSeeker resulted from a collaboration between industry and advocacy partners. Ayogo partnered with the Diabetes Hands Foundation (DHF), who provided creative vision and execution for the project. The Joslin Diabetes Center of the Harvard Medical School developed and reviewed all medical content. The project was supported by Boehringer Ingelheim Pharmaceuticals, Inc.

HealthSeeker combined a supportive social networking environment with important information on managing diabetes for adults living with diabetes or who are prediabetic. The HealthSeeker game was also localized and translated to Spanish to engage Hispanic players, under the name Explorando tu Salud Paso a Paso. Game players chose missions to help them achieve specific lifestyle goals, such as eating healthier, achieving an optimal weight, or lowering blood sugar levels.

HealthSeeker was designed with the Transtheoretical Model [35] in mind. Gamification elements were strategically employed to motivate the player as they pass through Stages of Change. Primary research by the partners determined that a diagnosis of diabetes is often accompanied by a sense of loneliness. This insight prompted a social design. The game drew upon the player’s social graph—their network of Facebook friends—to motivate repeat engagement with the technology. People with type 2 diabetes used the game to progress from contemplation of necessary lifestyle changes to preparation and action. The game was structured around small actionable missions developed by clinicians at the Joslin Diabetes Center. All features were social, including the ability to challenge and share the mission with others, share and celebrate by tweeting and posting accomplishments, and player-to-player gifting of virtual kudos for achievements.

The app was promoted to diabetes educators as an innovative patient engagement tool for patients who are enticed by technology and motivated by more timely feedback from other users. A distinct advantage of this technology is the immediate reward of positive feedback for behavior change efforts. Educators may also find that patients are better able to make choices from a variety of examples that are realistic, measurable, and achievable in the short term, which may subsequently lead to longer-term rewards. The use of words like “mission” and “kudos” instead of “behavioral goals” or “objectives” adds a softer, if not clearer, definition of what is expected of the patient. Educators also appreciate that the HealthSeeker examples are easily translated into action steps for people without diabetes as well, making the behavior more realistic to implement [36].

Before the game was retired, nearly 20,000 players had tried HealthSeeker—more than 3700 completed missions and more than 42,000 healthy actions were taken, including 20,500 healthy meals eaten (source: Facebook app statistics). HealthSeeker won several awards and generated over 83 original news stories/blog posts and over 275 million media impressions (source: Ayogo statistics).

The Joslin Diabetes Center did a qualitative evaluation of the game and found players responded positively to the game and were motivated to achieve appropriate blood glucose levels through appropriate diet, increasing exercise, strengthening social support, and reducing stress [36].

**Figure 4:**
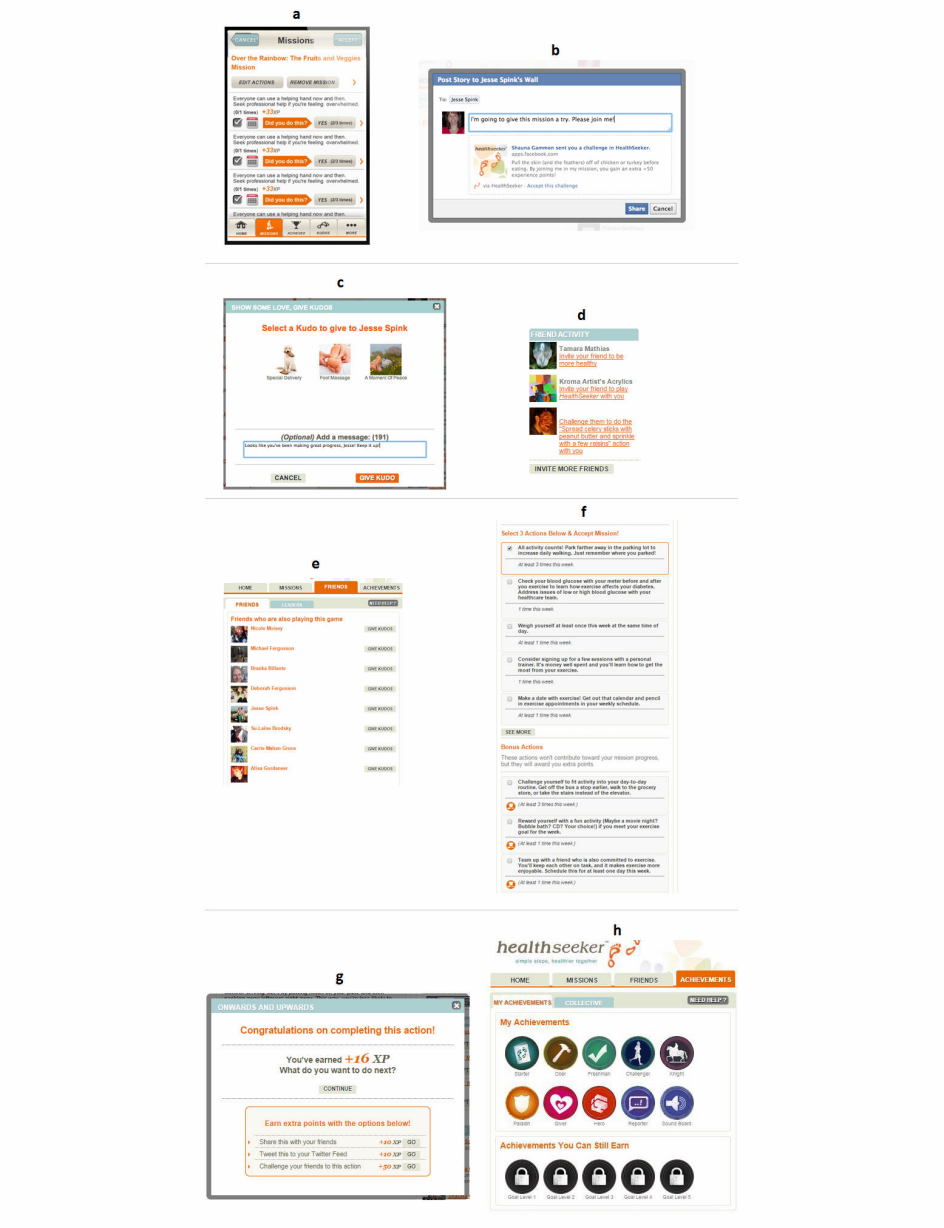
User-centered metrics analyzed in HealthSeeker: (a) Missions, (b) Challenges, (c) Kudos, (d) Invitations, (e) Friends, and (f) Actions. Additional HealthSeeker features are shown in (g) earning XP and social media sharing of players’ accomplishments, and (h) Achievement Badges.

Klauser et al [37] looked at social player analytics in HealthSeeker to understand how socially-engaging gameplay behavior influenced player interaction. Their results showed that actively social players solved more missions than players without friends. They concluded that a “well-connected social network can improve a user’s success to solve health missions and therefore help to live healthier.” The core engagement metric for HealthSeeker was how many healthy missions were completed. User-centered metrics that were analyzed included:

1. Missions: A mission is a small collection of repeatable health actions that must be accomplished within a set time period. For example, Over the Rainbow: The Fruits and Veggies Mission. See Figure 4, part a.

2. Challenges: Peer-to-peer requests to participate in a mission. See Figure 4, part b.

3. Kudos: A virtual gift with encouraging message. See Figure 4, part c.

4. Invitations: See Figure 4, part d.

5. Friends: See Figure 4, part e.

6. Actions: See Figure 4, part f.

The average number of missions players completed within the first 2 months of play was 6. Players who sent at least one challenge from a friend completed twice the average, or 12 missions. Players who received at least one challenge—whether they accepted the challenge or not—completed, on average, 18 missions. Social elements that allowed players to send and receive challenges increased player engagement.

The key insight of “the power of the incoming message” is that design can foster peer comments, “likes,” and encouragements, which are seen in HealthSeeker as effective at stimulating and sustaining intrinsic motivation to build good health habits. This design principle is now applied by Ayogo wherever possible in other health game designs.

HealthSeeker illustrates how various game mechanics can be applied in a complementary and effective way within a single social game, offering feedback through game points, completion status, progress bars, and automated congratulatory messages. Game mechanics can also offer sociability in the form of peer-to-peer challenges and messaging, urgent optimism through immediate challenge, plus perceived high likelihood of success (eg, “You can do this now”). Other feedback mechanisms are progressive mastery (ie, as the player progresses, they “level up” to harder challenges), loss aversion (ie, strive to retain points—failing to complete within time frame halves points), virality (ie, when completed, a pop-up reward appears giving experience points, with option to “Tweet this” or Facebook “share” or “challenge a friend”, all for bonus experience points—see Figure 4, part g), player status based on experience points in game, Achievement Badges (see Figure 4, part h) that can be collected, and reciprocity and repeat engagement encouraged by gifting of Kudos.

## Discussion

### On Gamification and Its Underpinning Principles

**Figure 5:**
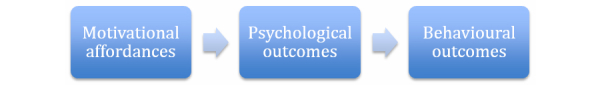
Gamification features (adapted from Hamari et al [38]).

Conceptualizing gamification for healthy behavior change, Hamari et al [38] simplified it into three components: motivational affordances, psychological outcomes, and behavioral outcomes (Figure 5). Motivational affordances include the following game features: points, leaderboards, achievements/badges, levels, stories, goals, feedback, rewards, progress, and challenges. Psychological outcomes include motivation, attitude, and enjoyment, while behavioral outcomes include whatever intended behavior was targeted, such as healthier eating, monitoring blood glucose levels, or exercising for a specific amount of time or intensity [38,39]. Gamification features are not dissimilar to validated behavior change elements, including providing feedback on performance, goal setting (ie, self-efficacy), and comparison with others, to name a few [40].

Self-efficacy is an important concept to understand when building tools that support people with diabetes in managing their disease [41]. In his book on social cognitive theory, Bandura [42] explains that self-efficacy is a prognostic factor for increasing healthy behaviors. Bandura identified four sources at the heart of self-efficacy: (1) one’s personal experience of success or failure, (2) vicarious experiences—observing others’ success or failure, (3) verbal or social persuasions, such as messages people receive from peers and family members, and (4) physiological and affective states. Improving self-efficacy has been shown to improve health outcomes for adults with type 2 diabetes and obesity, as well as for children and teenagers with type 1 diabetes.

Designs for people with diabetes should, therefore, build self-efficacy by (1) providing a series of progressive, small successes or achievements related to diabetes management, (2) creating vicarious experience through social features that allow peer-to-peer sharing of successes and tips on how to manage diabetes, (3) maximizing incoming social messages of encouragement, and (4) providing empowering and empathic messages and narratives.

A design approach to health games that is consistent with both Bandura [42] and Krichbaum et al [41], and touches on the above four sources of self-efficacy would be expected to be helpful in diabetes self-management.

The Ayogo Model presented earlier in this paper incorporates an understanding and application of self-efficacy [41,42], cognitive biases [43,44,45,46], persuasive technology (ie, captology [47,48]), and empathy-triggering mechanisms via oxytocin-releasing actions (ie, human oxytocin-mediated empathy [HOME]) [49,50]. HealthSeeker and the other games presented in this paper offer examples of how gamification should and can go beyond Points, Badges, and Leaderboards (PBL) in order to effect the desired health and behavior-changing outcomes [51].

Another important and overlapping concept is self-regulation, or the ability to control our own behavior, which can be described as the process involved in the continuous pursuit of goals and in dealing with different obstacles along the way. Self-regulation is construed as a systematic process that involves conscious efforts to influence thoughts, feelings, and behaviors, in order to achieve a goal in the context of a changing environment or challenging disease condition [52]. Providers of medical assistance to diabetes patients should positively present monitoring (ie, measuring blood glucose) as a *measure of performance* as opposed to a measure of disease (ie, diabetes) [53]. This has implications for the design of games in diabetes. Mainstream video games have long similarly measured and motivated players’ performance. To achieve this, the games often have a tracking system and feature some in-game goals. Games can motivate both extrinsically (eg, via use of in-game rewards and recognition) and intrinsically (eg, by nurturing those conditions leading to a sense of autonomy and competence by the player/patient).

### Some Concerns and Limitations of the Presented Games

Gamified interventions could have limitations influenced by users’ age, culture, clinical state, digital-/health-literacy levels [54], and/or other relevant personal traits. According to Gartner, the vast majority of gamification projects taking place today are lacking in some respect and thus “run the risk of falling into disuse, once their novelty wears off” [55]. The mere application of video game concepts to the health arena must take into consideration all clinical and individual users’ aspects of the health issues to be addressed. As well, without researching and applying the pertinent evidence-based psychological knowledge of behavioral change strategies, the credibility of this class of solutions and users’ long-term adherence to them will be put in danger. That is why the authors believe that more evidence, guidelines, and validated models are needed to support the application of gamification to diabetes and other medical conditions, and to ensure its long-term success, wide adoption, and sustainability.

Exercise is highly recommended by diabetes clinicians [56]. Monster Manor (for type 1 diabetes) does not have exercise guidelines. HealthSeeker did have activity-related content. But the difficulty of managing blood glucose before, during, and especially after exercise should be carefully considered in future game apps of this type, whenever in-app information or guidelines are offered to patients regarding exercise.

The difficulty in managing childhood diabetes occurs largely during school time, which forms a large proportion of the day and is a period during which the child is not supervised by the parent. During the pilot test of Monster Manor, Ayogo discovered that the app was not usable at school for most children in the test population. Future iterations would revise the use case to accept this difficulty—that many children are currently limited to playing the game on their parents’ mobile phones outside school time.

Achieving “optimum” diabetes control can lead to a heightened risk of hypoglycemia. Designers of game apps should be mindful of this when attempting to influence a patient’s self-management, to avoid iatrogenic (ie, app-induced) hypoglycemia, although this has not been reported so far with any of the presented games. The team of designers should always include in its membership qualified diabetologists to clinically inform and guide the development of these games, which is the approach already adopted by Ayogo.

### Conclusions

This paper provided a brief taster of some of the games on offer for people with type 1 and type 2 diabetes belonging to different age groups. This paper was not meant to serve as an exhaustive survey of all available digital games for diabetes from different providers, nor as a formal summative evaluation of this range of games (or of any individual game example). However, we believe our paper has provided enough “food for thought” to appreciate the main challenges facing people with diabetes—children and adults—in coping with their lifelong condition. We have highlighted and showcased some of the potential benefits to be gained from deploying gamified digital interventions and platforms as adherence tools, and more, in diabetes. Further research evidence from full-scale summative evaluation studies of existing games is needed, and indeed expected in the near future, that will quantify, qualify, and establish the evidence base concerning this gamification potential and how to best harness it, such as what works in each age group/patient type, what does not, and under which settings and criteria. Frameworks such as the one described in Graafland et al [57] will prove helpful in gathering this evidence in a consistent way.

## Abbreviations

DHF: Diabetes Hands Foundation
GPS: global positioning system
HOME: human oxytocin-mediated empathy
PBL: Points, Badges, and Leaderboards
UI: user interface
UX: user experience
XP: experience points
